# Protection of procyanidin B2 on mitochondrial dynamics in sepsis associated acute kidney injury via promoting Nrf2 nuclear translocation

**DOI:** 10.18632/aging.103726

**Published:** 2020-08-15

**Authors:** Jian-Xing Liu, Chen Yang, Ze-Jian Liu, Hong-Yong Su, Wei-Huang Zhang, Qingjun Pan, Hua-Feng Liu

**Affiliations:** 1 Key Laboratory of Prevention and Management of Chronic Kidney Disease of Zhanjiang City, Affiliated Hospital of Guangdong Medical University, Zhanjiang 524001, Guangdong, China; 2 Guangdong Medical University, Zhanjiang 524023, Guangdong, China

**Keywords:** septic acute kidney injury, mitochondrial dynamics, reactive oxygen species, procyanidin B2, Nrf2 pathway

## Abstract

In septic acute kidney injury (SAKI), the positive feedback between damaged mitochondria and accumulation of reactive oxygen species results in cell and tissue damage through multiple mechanisms. Removing the damaged mitochondria or neutralizing the reactive oxygen species has been considered beneficial to alleviating cell damage. The antioxidant Procyanidin B2 has been reported to inhibits reactive oxygen species and thereby reduces cell injury. However, it is unclear whether this effect is associated with clearance of damaged mitochondria. Here, we evaluated the efficacy of procyanidin B2 on SAKI, and focused on its effects on mitochondrial dynamics and removing damaged mitochondria via mitophagy. The results showed that the renal function, renal tubular cell vacuolization and oxidative stress were decreased in SAKI mice treated with procyanidin B2, moreover, skewed mitochondrial fusion/fission, mitochondrial mediated apoptosis and impaired mitophagy were improved in SAKI mice treated with procyanidin B2. In mechanism, the improvement of procyanidin B2 on mitochondrial dynamics were associated with increased nuclear translocation of the transcription factor, Nrf2. In summary, our findings highlighted that the protective efficacy of procyanidin B2 in reducing cellular damage in SAKI, and mechanisms improving mitochondrial dynamics and quality control at least in part by promoting Nrf2 translocation into the nucleus.

## INTRODUCTION

Sepsis is among the most common causes of acute kidney injury (AKI) and accounts for approximately 50% of all AKI cases [1]. The mortality of patients who suffer from sepsis with AKI is 30–60%, which is approximately two-fold of that of patients without AKI [2, 3]. In recent years, the critical role of mitochondria in renal tubular epithelial cell pathophysiology in septic AKI (SAKI) has been confirmed that the renal tubular epithelial cell damage is closely associated with mitochondrial damage and reactive oxygen species (ROS) [4, 5]. Researchers found that the vacuolization of tubular epithelial cell and decrease level of ATP were mainly occurred at sites that with the densest load of mitochondria and higher energy turnover, such as proximal convoluted tubule and the thick ascending limb of Henle's loop segment [6, 7]. Mitochondria are the cells' power plants, and the damaged mitochondria are the main source of intracellular ROS, meanwhile, mitochondria are more vulnerable to intracellular ROS than the other organelles. In cellular, ROS was critical trigger of mitochondria damage [5, 8], and subsequently results in renal tubular epithelial cells damage through multiple mechanisms in SAKI [9, 10]. Therefore, neutralization of ROS or removing the damaged mitochondria is an important strategy to alleviate cell or tissue damage.

Procyanidin B2 (PB2), a typical representative of antioxidants, belonging to the class of proanthocyanidin flavonoid polyphenols, are widely distributed among plants such as grape seed, berry fruits, cocoa, and tea, and possesses a broad spectrum of pharmacological properties, such as anti-oxidative and anti-inflammatory effects [11–13]. Previous studies have reported the beneficial effects of PB2 on high glucose-induced mitochondrial dysfunction in podocytes and mesangial cells [14, 15], and on promoting lysosomal degradation of substances in hepatic steatosis [16]. However, it remains unknown whether these beneficial effects associated to ameliorates mitochondria damage and improves impaired mitophagy in severe stress condition. Thus, this study aimed to investigate whether PB2 can reduce damaged mitochondria and ROS accumulation-induced cell damage by promoting the clearance of damaged mitochondria in SAKI. In the present study, the protective effects of PB2 on SAKI was evaluated in an LPS-challenged mouse, and the relationship between renal protection of PB2 and the improvement of mitochondrial dynamics and inhibition of ROS was explored.

## RESULTS

### Effects of PB2 on pathological changes and renal function

**Figure 1:**
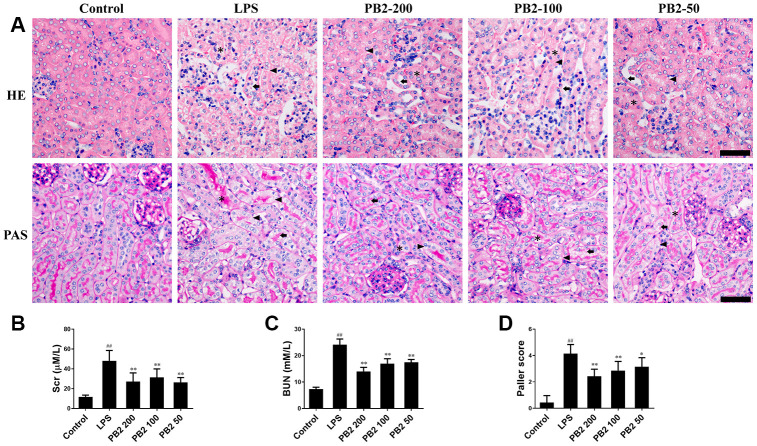
**Effects of PB2 on pathological changes and renal function (n = 8).** Mice were challenged with 10 mg/kg LPS (i.p.) and treated with PB2 (i.g.). 24 h after LPS challenged, mice were sacrificed. HE and PAS (Bar = 50 μm; asterisks: cast, arrowhead: vacuolization, arrow: loss of brush border) (**A**) and Quantification of tubular injury (**D**); Serum creatinine (**B**), Blood urea nitrogen (**C**). ^#^P < 0.05, ^##^P ≤ 0.01 compared to control mice; ^*^P ≤ 0.05, ^**^P ≤ 0.01 compared to LPS challenged alone.

First, the protective effect of PB2 on pathological damage and renal function in SAKI mice were evaluated. The morphological results showed that renal tubular epithelial cell vacuolization occurred in most of the tubule, and was accompanied with scattered renal casts and detached cells in the renal tubular lumen in SAKI mice. In contrast, these pathological damages, especially the extent of vacuolization in tubular epithelial cells, were reduced significantly SAKI mice kidney that treated with PB2 (Figure 1A and 1D). Furthermore, the elevated levels of Scr and BUN were also decreased in PB2 treated SAKI mice kidney (Figure 1B and 1C).

### Effects of PB2 on mitochondrial structure and mitochondria-mediated apoptosis

**Figure 2:**
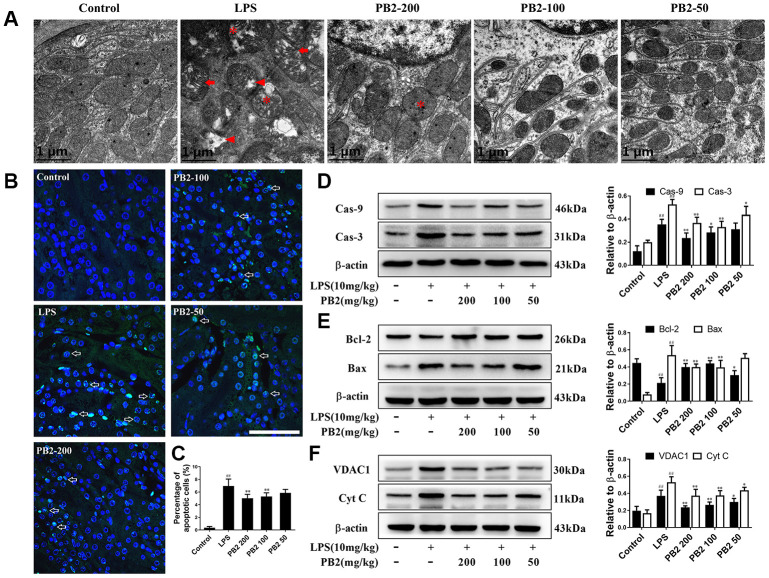
**Effects of PB2 on mitochondrial structure and mitochondrial-mediated apoptosis (n = 8).** Mitochondrial structure in renal tubular epithelial (Bar = 1 μm) (**A**); TUNEL staining (Bar = 50 μm, green fluorescent spots) (**B**), and percentage of TUNEL-positive cells (**C**); expressions of caspases, Bcl-2, Bax, VDAC1, Cyt c and the semi-quantification (**D**–**F**). ^#^P ≤ 0.05, ^##^P ≤ 0.01 compared to control mice; ^*^P ≤ 0.05, ^**^P ≤ 0.01 compared to LPS challenged alone.

We next evaluated whether the protective effect of PB2 involved reducing mitochondrial damage. TEM was used to evaluate the potential protective effects of PB2 on mitochondria in kidney tubular epithelial cells. The results showed that mitochondria damage that characterized by swelling, cristae widening and breakage, and disintegration were extensive occurred in SAKI mice renal tubular epithelial cells, especially in those of the proximal tubular cells. However, these mitochondria damages were mitigated in the PB2-treated SAKI mice kidney, mitochondrial disintegration was rare, the swelling, cristae widening and breakage were decreased (Figure 2A). As damaged mitochondria are involved in apoptosis of tubular epithelial cells via many intracellular signaling pathways, thus the effects of PB2 on mitochondria-mediated apoptosis was evaluated. The results showed that the apoptotic positive cell was decreased in PB2-treated SAKI mice kidney (Figure 2B and 2C), the expression of pro-apoptotic proteins of caspase-9 and caspase-3, which can be induced by mitochondrial damage, were also decreased in PB2-treated SAKI mice kidney (Figure 2D). Furthermore, the expression levels of Bax, VDAC1, and cytochrome c, which are marker proteins that respond to mitochondrial damage and play an important role in apoptosis, were also significantly decreased, whereas the level of Bcl-2 was obviously increased in PB2-treated SAKI mice kidneys (Figure 2E and 2F). Together, these results suggest that PB2 could reduce mitochondrial damage and mitochondrial-damaged mediated apoptosis.

### Effects of PB2 on mitochondrial dynamics

**Figure 3:**
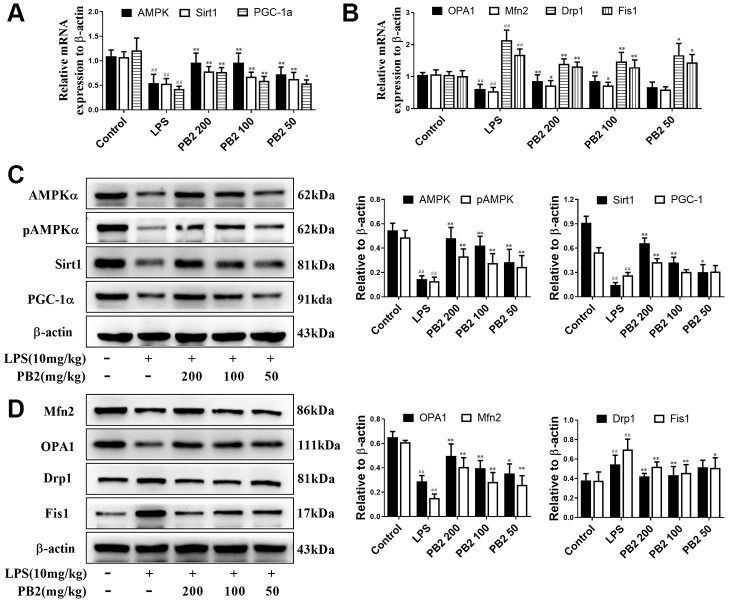
**Effects of PB2 on mitochondrial biogenesis and fusion/fission (n = 8).** The mRNA of AMPK, Sirt1 and PGC-1α (**A**), OPA1, Mfn2, Drp1 and Fis1 (**B**); expressions of AMPK, Sirt1, PGC-1α, OPA1, Mfn2, Drp1 and Fis1, and semi-quantification (**C**, **D**). ^#^P ≤ 0.05, ^##^P ≤ 0.01 compared to control mice; ^*^P ≤ 0.05, ^**^P ≤ 0.01 compared to LPS challenged alone.

Given that mitochondrial excessive fission or fragmentation is a critical trigger for apoptosis via releasing cytochrome C from mitochondria to cytoplasm, thus the effect of PB2 on mitochondrial dynamics was evaluated in followed. Polymerase chain reaction (PCR) and western blotting results showed that the mitochondrial dynamics were aberrant, which characterized by decreased biogenesis and skewed to fission in SAKI mice kidneys. Notably, the disturbed mitochondrial dynamics was reversed in PB2-treated SAKI mice kidney. The expression of AMPKα, Sirt1, PGC-1α that major function in mitochondrial biosynthesis were increased, expression of OPA1 and Mfn2 that role in mitochondrial fusion were also increased, whereas the expression of Drp1 and Fis1, which are involved in mitochondrial fission, were decreased in PB2-treated SAKI mice kidney, whether in the gene or protein level (Figure 3A–3D).

### Effects of PB2 on mitophagy

**Figure 4:**
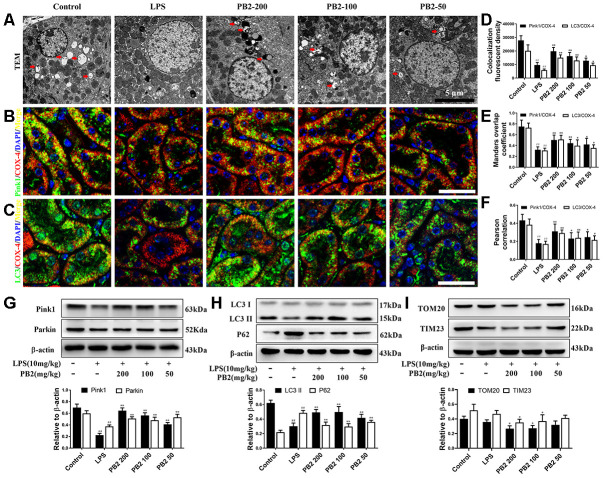
**Effects of PB2 on mitophagy (n = 8).** Mitochondrial structure in renal tubular epithelial (Bar = 5 μm) (**A**); co-localization of anti-LC3 (green)/anti-COX IV (red) antibodies (**B**) and anti-Pink1 (green)/anti-COX IV (red) antibodies (**C**) (Bar = 25 μm); the co-localization fluorescence intensity, overlap coefficient and Pearson's correlation of LC3-COX IV and Pink1-COX IV (**D–F**); expression of Pink1, parking, LC3 and p62, and semi-quantification (**G–I**). ^#^P ≤ 0.05, ^##^P ≤ 0.01 compared to control mice; ^*^P ≤ 0.05, ^**^P ≤ 0.01 compared to LPS challenged alone.

The effects of PB2 on mitophagy was investigated in followed, and the results showed that mitophagy was decreased in SAKI mice kidney, while the impaired mitophagy was pick-up in PB2-treated mice kidney. The TEM results showed that the level of mitophagy was pick-up after treated with PB2 (Figure 4A). Subsequently, we evaluated the characteristic events that representing the early and mid-phase of mitophagy by co-localization of Pink1/COX IV and LC3/COX IV. The results showed that both the co-localization fluorescent density, overlap coefficient and Pearson's correlation of Pink1/COX IV and LC3/COX IV were increased in PB2-treated SAKI mice kidney (Figure 4A–4F). Additionally, the levels of LC3, Pink1, and Parkin were as well as increased in PB2-treated SAKI mice kidney, and accompanied by decreased levels of TOM20 and TIM23 (Figure 4G–4I).

### Effects of PB2 on ROS

**Figure 5:**
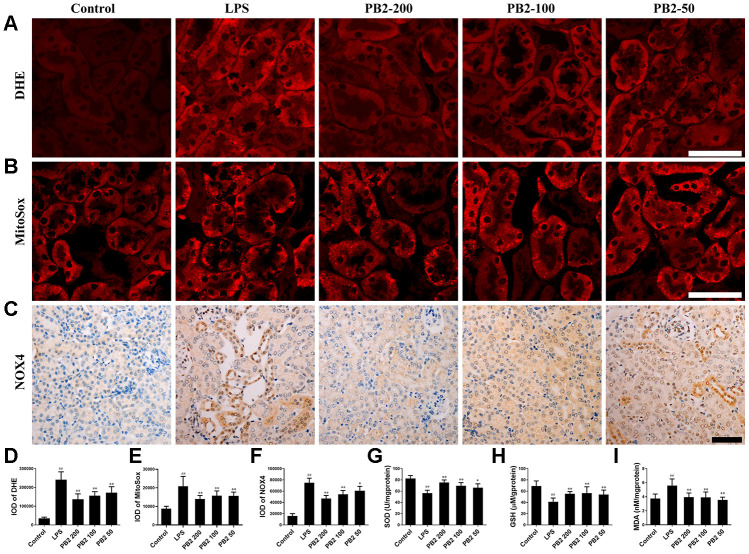
**Effects of PB2 on reactive oxygen species (n = 8).** DHE (**A** and **D**), MitoSox red (**B** and **E**), NOX4 (**C** and **F**) (Bar = 50 μm); The levels of SOD (**G**), reduce GSH (**H**) and MDA (**I**) in renal tissue. ^#^P ≤ 0.05, ^##^P ≤ 0.01 compared to control mice; ^*^P ≤ 0.05, ^**^P ≤ 0.01 compared to LPS challenged alone.

In cellular, ROS was the critical trigger of mitochondria damage and subsequently cells damage. Thus, the effect of PB2 on mitochondrial-derived ROS was assessed in further by DHE and MitoSox red. The results showed that the fluorescence intensity of DHE and MitoSox were increased significantly in SAKI mice kidney, whereas the level of superoxide (O^2-^) was significantly decreased in PB2-treated SAKI mice kidney (Figure 5A–5B and 5D–5E). Mitochondria are not the only source of ROS, and the NADPH oxidase (NOX) family, especially NOX4, also play an important role in ROS production in kidney [17]. Consistent with the results of O^2-^, the expression of NOX4 was also decreased in PB2-treated SAKI mice kidney (Figure 5C and 5F). In addition, the level of antioxidant enzyme, such as SOD and GSH, were increased, while the level of MDA was decreased in PB2-treated SAKI mice kidney (Figure 5G–5I).

### Effects of PB2 on Nrf2 translocation

**Figure 6:**
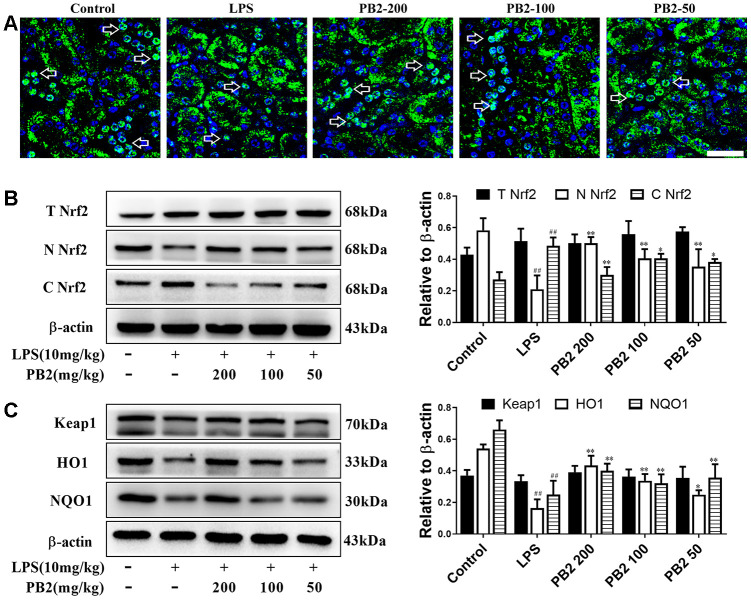
**Effects of PB2 on Nrf2 translocation into nucleus (n = 8).** IF of Nrf2 (Bar = 50 μm) (**A**); total Nrf2 and nuclear/cytosolic Nrf2 (**B**), Keap1, HO1 and NQO1 and semi-quantification (**C**). ^#^P ≤ 0.05, ^##^P ≤ 0.01 compared to control mice; ^*^P ≤ 0.05, ^**^P ≤ 0.01 compared to LPS challenged alone.

In view of the important role of ROS in the entire mitochondrial dynamics and the critical role of Nrf2 on ROS regulation, the inhibitory effect of PB2 on ROS and on improvement of mitochondria whether associated to translocation of Nrf2 into nuclear was further investigated. The results showed that the level of total Nrf2 and Keap1 without obviously change, however Nrf2 was prone to be localized in the cytoplasm rather than in nucleus in SAKI mice kidneys. Significantly, the level of Nrf2 in nuclear was increased in PB2-treated SAKI mice kidney, although the total level of Nrf2 and Keap1 were without obvious impacted by PB2 (Figure 6A and 6B). As well as, the phase II detoxifying enzymes, HO1 and NQO1, were also increased in PB2-treated SAKI mice kidney (Figure 6C).

### Effect of PB2 on mitochondrial dynamics depend on Nrf2 translocation

**Figure 7:**
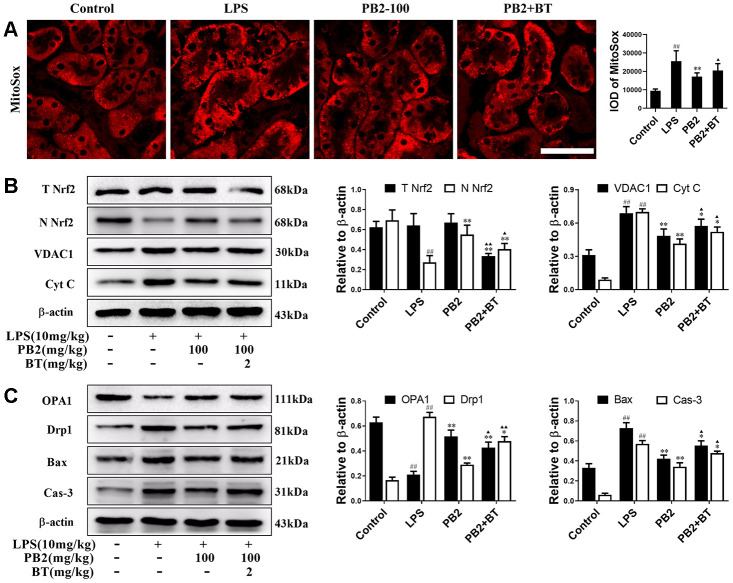
**Improved mitochondrial dynamics depend on Nrf2 translocation (n = 8).** Mice in co-treatment group were treated with brustatol 24h prior LPS challenge in i.p.. The mitoSox red (Bar = 50 μm) (**A**); expressions of total and nuclear Nrf2, VDAC1 and Cyt c (**B**), OPA1 and Drp1, Bax and caspase-3 and semi-quantification (**C**). ^#^P ≤ 0.05, ^##^P ≤ 0.01 compared to control mice; ^*^P ≤ 0.05, ^**^P ≤ 0.01 compared to LPS challenged alone; ^**▲**^P ≤ 0.05, ^**▲▲**^P ≤ 0.01 between PB2 and PB2+BT.

**Figure 8:**
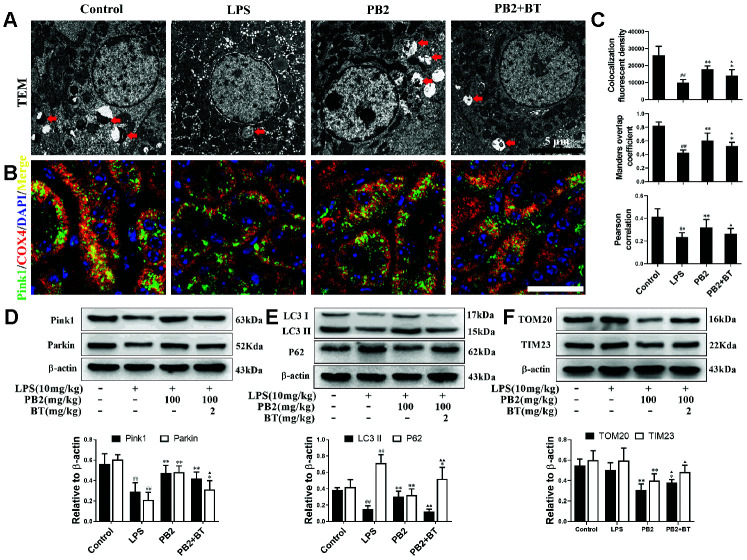
**Improved mitophagy depend on Nrf2 translocation (n = 8).** Mitochondrial structure in renal tubular epithelial (Bar = 5 μm) (**A**); co-localization of anti-Pink1 (green)/anti-COX IV (red) antibodies (**B**) (Bar = 25 μm); the co-localization fluorescence intensity, overlap coefficient and Pearson's correlation of Pink1-COX IV (**C**); expressions of Pink1, parking, LC3, p62, TOM20 and TIM23, semi-quantification (**D**–**F**). ^#^P ≤ 0.05, ^##^P ≤ 0.01 compared to control mice; ^*^P ≤ 0.05, ^**^P ≤ 0.01 compared to LPS challenged alone; ^**▲**^P ≤ 0.05, ^**▲▲**^P ≤ 0.01 between PB2 and PB2+BT.

Further, to determine whether the protective efficacy of PB2 on mitochondrial dynamics depended on Nrf2, an inhibitor of the Nrf2 transcription factor, brusatol (BT) was used [18]. Thirty-two male mice (approximately 7–8 weeks, weighing 18–22 g) were randomly divided into four groups (n = 8): control, LPS, LPS + PB2 (100 mg/kg), and LPS + PB2 (100 mg/kg) + BT (2 mg/kg). Mice in the PB2 and BT co-treatment group were treated with BT (i.p.) 24 h prior to the LPS challenge, and mice were sacrificed at 24 h after LPS challenge. Samples were then harvested and analyzed as indicated in the Methods section. The results showed that the protective effects of PB2 were weakened upon co-treatment with BT, the mitochondrial ROS (Figure 7A) and the levels of cytochrome c and VDAC1 (Figure 7B) were increased in co-treated SAKI mice kidney than those in PB2 alone treated mice kidney. Similarly, the expression of OPA1 was reduced and the levels of Drp1, Bax, and caspase-3 were elevated in co-treated SAKI mice kidney when compared with those in PB2 treated alone mice (Figure 7C). Furthermore, pick-up level of mitophagy by treatment of PB2 was blunt in co-treated SAKI mice kidney (Figure 8A), the fluorescence intensity and co-localization of Pink1/COX4 were also fall back in co-treated SAKI mice kidney (Figure 8B and 8C). As well as, the levels of Pink1, Parkin, and LC3 (Figure 8D and 8E), and the levels of TOM20 and TIM23 were decreased in the co-treated mice kidney (Figure 8F).

## DISCUSSION

In SAKI, the most vulnerable damage segment was the S2 and S3 proximal tubular, which the segment with the most efficient filtration and re-absorption and the most density of mitochondria. The proximal tubular epithelia cells damage were characterized by the extensive vacuolization and scattered apoptosis, more importantly the decreased levels of ATP and outbursts ROS were occurred before these damage [19]. These suggest that mitochondria, as the cells' power plants and main source of ROS organelle, was injured in these segments, and take critical part in tubular cell damage in SAKI [20, 21]. Mitochondria was the main source of ROS in cellular, however, in turn they are more vulnerable to ROS than other organelles, and could initiate inflammation and apoptosis via multiple mechanisms once changes either in morphology or in function [8, 9, 22]. More importantly, the positive feedback between damaged mitochondria and ROS initiates a vicious circle that further amplifies the cell damage in SAKI [23].

Studies have found that ROS exerts multiple effects on mitochondria, including mitochondrial damage, mitochondrial fusion/fission, mitochondrial-mediated apoptosis and mitophagy [24, 25]. Excessive accumulation of ROS changes in mitochondrial membrane potential and permeability, and subsequently results in mitochondria prone to fission to separates low-potential part and reserve normal-potential part mitochondria [26–28]. Meanwhile, changes in mitochondrial membrane potential and permeability leads to the leakage of cytochrome C from mitochondria to cytoplasm, and subsequently initial apoptosis. Significantly, use of antioxidants to suppress ROS outburst beneficial to improves mitochondrial dynamics and reduces mitochondrial damage or cell apoptosis in SAKI [29–32]. Mitochondrial dynamics are precisely regulated by the fusion and fission proteins in the inner/outer membranes of mitochondria, such as Mfn2/OPA1 (mediates fusion) and Fis1/Drp1 (mediates fission), and are important for mitochondrial quality control, energy homeostasis, and protecting against cell damage [33]. Generally, at least one of the daughter mitochondrion that splits from damaged mitochondria shows low-potential, loses the ability to fuse with normal mitochondria, and is prone to degradation [34]. This specific degradation of damaged mitochondria is defined as mitophagy, and is mainly regulated by the Pink1-Parkin signaling pathway [35]. Briefly, the reduction of the inner and outer membrane potential gradients causes Pink1 to aggregate on the outer mitochondrial membrane rather than being transported into the inner mitochondrial membrane for degradation. This is followed by recruitment of Parkin, which interacts with the ubiquitin-binding factor p62, and subsequent binding to LC3 to promote encapsulation of mitochondria and degradation by lysosomes [36–38]. Thus, mitophagy is a self-protective cellular response to remove damaged or nonfunctional mitochondria; however, this protective process can be impaired under overwhelming cellular stress or when the numbers of damaged mitochondria are beyond the threshold capacity for degradation [39–41]. Impaired mitophagy has been observed along with the appearance of cell damages in SAKI [42, 43], and it was closely associated with excessive mitochondrial ROS, release of pro-apoptotic proteins and initiation of mitochondrial apoptotic cascade, and subsequent cell injury and death [44]. The protective effect of mitophagy through reducing the accumulation of damaged mitochondria and ROS have been demonstrated in numerous researches [24, 45]. Loss of mitophagy worsens kidney damage, while promoting mitophagy reduces mitochondrial damage as well as ROS accumulation [44, 46, 47]. Meanwhile, the leakage of cytochrome c, Ca^2+^, and mtDNA that occurs in the absence of timely clearance of damaged mitochondria can activate NLRP3 and caspases [48, 49], and subsequently inhibits mitophagy via direct degradation of Parkin [34, 35]. Importantly, the interplay between damaged mitochondria, ROS, and mitophagy could be more complicated in severe stress conditions. But overall, decreasing the accumulation of damaged mitochondria through mitophagy or inhibiting excessive intracellular ROS accumulation is considered as an effective strategy for reducing cell damage [44, 50–52].

The anti-inflammatory and anti-oxidant effects of PB2 have been proven in many studies. Although ROS is an important trigger for mitochondrial damage and plays a role in initiation of mitophagy, excessive ROS may overwhelm the process of clearance of damaged mitochondria. Therefore, it remains unknown whether the increased mitophagy observed after PB2 treatment was due to neutralization of ROS by antioxidant enzymes, or due to the direct activation of mitophagy. Recently, important effects of PB2 on mitochondrial morphology, oxidative phosphorylation, and mitochondrial-mediated apoptosis were found [12, 53]. In addition to anti-oxidant effects, the effects of PB2 on improving mitochondrial function were found. Research found that PB2 beneficial to reduces mitochondrial superoxide radical and mitochondrial membrane potential loss and mitochondrial-mediated apoptosis, meanwhile increases mitochondrial content, and promotes ATP production via increasing oxygen consumption rate and restoring the damaged mitochondrial respiratory capacity of oxidative phosphorylation [12, 15, 53–56]. However, these findings did not rule out the possibility that the effect of PB2 on inhibiting mitochondrial damage was due to indirectly inhibiting excessive ROS. Additionally, recent research found that in free fatty acids-induced hepatic steatosis, PB2 promotes lysosomal-related degradation [16], a necessary process for damaged mitochondrial degradation in later phase of mitophagy, further suggesting that PB2 may also have a role in promoting the clearance of damaged mitochondria. In addition, considering that ROS is an inducer of mitophagy, the inhibition of ROS by PB2 may reduce the mitophagy flux. Moreover, if the accumulation of damaged mitochondria is reduced due to the reduction of ROS, then PB2 should cause a consistent decrease in ROS and mitophagy. However, our results show that PB2 inhibits ROS and increases mitochondrial autophagy, which suggests that excess ROS in SAKI may inhibit the mitophagy flux or that there are other mechanisms involved in promoting mitophagy.

Studies have shown that the effect of PB2 on oxidative stress is associated with increased Nrf2 translocation into the nucleus [57–59], and similar effects were found in this study. Nrf2 is the master antioxidant transcription factor and its stability is suppressed through the action of the cytoplasmic Cullin3KEAP1 ubiquitin ligase complex under conditions of homeostasis [60]. Under oxidative stress, KEAP1, a cysteine-rich protein, acts as a redox sensor, and the oxidative modification of critical cysteines of KEAP1 leads to dissociation of Nrf2-KEAP1 from CUL3 thereby preventing Nrf2 degradation [61]. Stabilization of Nrf2 allows for its translocation into the nucleus where it induces the expression of a battery of Phase II antioxidant and detoxification genes, after binding to the antioxidant response promoter elements (ARE). In addition, PB2 can phosphorylates Nrf2 [57], and the phosphorylation of Nrf2 is known to stabilize the protein and promotes its nuclear translocation [62, 63]. In this study, the Nrf2 nuclear translocation was found increased in SAKI mice kidney that treated with PB2, and the Phase II antioxidant, such as SOD, CAT, HO1 and NQO1, were gone up accordingly. Meanwhile, the translocation of Nrf2 into nucleus was beneficial to improves overall mitochondrial homeostasis [64], knockout of Nrf2 or suppression of Nrf2 by siRNA results in decreased in mitochondrial number, mitochondrial content, mtDNA copy number [65–67], and an impaired mitochondria morphology were found in Nrf2-deficient mice, and along with decreased expression levels of genes involved in mitochondrial biogenesis and dynamics [68, 69], and the underlying mechanism was found to be associated with activation of the AMPK-SIRT1-PGC-1α axis [14, 15, 65, 66]. Significantly, as a sensor of bioenergy and upstream molecule of PGC-1α, pAMPK, plays an important role in the mitochondrial biogenesis network and has a direct role in the translocation of Nrf2 [70]. Therefore, co-activation of Nrf2 and pAMPK by PB2 and their combined function may contribute to mitochondrial biogenesis and redox homeostasis. Meanwhile, researchers found that Nrf2 has a direct role in mitophagy via direct binding to the ARE sites of Pink1 promoter, thereby upregulating the transcriptional expression of Pink1 and promoting mitophagy [71–73]. Therefore, we speculated that the effects of PB2 on improves of mitochondrial dynamics and mitophagy, and on reduces mitochondrial damage-mediated apoptosis were not only due to the indirect effect of Nrf2 nuclear translocation on inhibiting ROS accumulation, but also including the directly translocation of Nrf2 on mitochondria biogenesis and mitophagy. In this study, the level of nuclear Nrf2 was decreased in PB2 and brusatol co-treatment mice kidney, the inhibition of mitochondrial ROS and of mitochondrial apoptosis were blunt, as well as blunt the improvement in mitochondrial dynamics. Although these changes could partly attribute to the Nrf2 nuclear translocation on ROS accumulation, however, it is worth noting that the directly effects of Nrf2 that promoting by PB2 on mitophagy was also found. The level of key protein Pink1 that initiation for mitophagy was decreased in co-treatment mice kidney, as well as mitophagy level.

**Figure 9:**
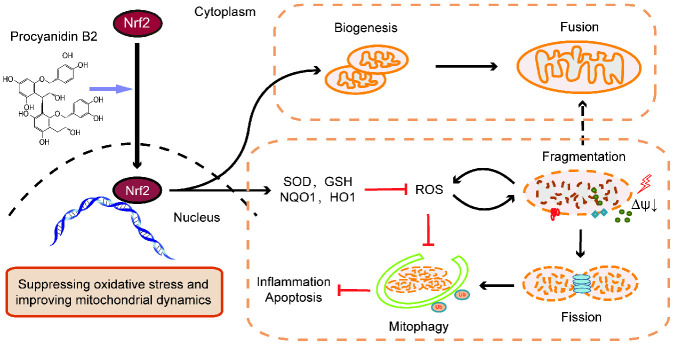
**Graphitic abstract**. The renal protective effects of PB2 was associated with promoted Nrf2 translocation into the nucleus, including directly effects on mitochondrial biogenesis and mitochondrial function and indirectly effects on improving mitochondrial dynamics and mitophagy via suppression of ROS accumulation.

In this study, multiple mechanisms underlying the protective efficacy of PB2 on LPS-induced SAKI were revealed. On one hand, Nrf2 nuclear translocation alleviates ROS accumulation and reduces mitochondrial damage. On the other hand, PB2 promotes mitochondrial biogenesis and improves mitochondrial dynamics directly via promoting Nrf2 translocation into the nucleus Figure 9. Meanwhile, the increased mitophagy also reduces the accumulation of damaged mitochondria and associated apoptosis and cell damage. Thus, promoting Nrf2 translocation into the nucleus is one of the important mechanisms underlying the protective effects of PB2 on SAKI.

## MATERIALS AND METHODS

### Mice

Specific pathogen-free (SPF) male C57BL/6 mice, weighing 18.0–22.0 g (approximately 7–8 weeks of age), were purchased from Guangdong Medical Laboratory Animal Center (SCXK-(Guangdong) 2013-0002). The mice were individually acclimated in ventilated cages in an SPF laboratory for 1 week prior to the experiments. The room was maintained at a temperature of 22 ± 2 °C, with 50 ± 10% relative humidity and a 12-hours light/dark cycle. Food and water were provided ad libitum. All animal experiments were approved by the Laboratory Animal Services Center at Guangdong Medical University (Zhanjiang, China) (SYXK-(Guangdong) 2015-0147) and performed according to the guidelines of Animal Welfare and Ethics of the Institutional Animal Care and Use Committee. All experiments were carried out in a specific pathogen-free animal lab.

### Materials

PB2 (purity≥95%) was obtained from Shanghai Yuanye Bio-Technology Co., Ltd (Shanghai, China), LPS (Escherichia Coli O111:B4) and Brusatol (purity≥95%) were obtained from Sigma (St. Louis, MO, USA). The primers were synthesized by Sangon Biotech Co., Ltd. (Shanghai, China), and primer sequences are shown in Table 1. A Simply total RNA extraction kit was purchased from Hangzhou Bioer Technology Co., Ltd. (Hangzhou, China). The reverse transcription kit and Q-PCR kit were obtained from Takara (Shiga, Japan). Deadend ^TM^ Fluorometric TUNEL system was purchased from Promega (Madison, WI, USA). The Serum Creatinine (Scr) kit, Blood Urea Nitrogen (BUN) kit, superoxide dismutase (SOD) assay kit, malondialdehyde (MDA) assay kit and, reduced glutathione (GSH) assay kit were purchased from Nanjing Jiancheng Bioengineering Institute (Nanjing, China). Dihydroethidium (DHE) and MitoSox Red Mitochondrial Superoxide Indicator (MitoSox) were obtained from Life Technologies (Carlsbad, CA, USA). Antibodies against Sirt1, AMPKα, p-AMPKα, Pink1, Parkin, and Bax were purchased from Cell Signaling Technology (Danvers, MA, USA), PGC-1α, OPA1, Drp1, Fis1, Nrf2, NQO1, HO1, VDAC1, caspase-9, caspase-3, Bcl-2, LC3, and P62 were purchased from Abcam (Cambridge, UK). TIM23 was purchased from Proteintech (Chicago,IL, USA), TOM20 and Cytochrome c were purchased from Santa Cruz Biotechnology (Dallas, TX, USA), Mfn2, Keap1 were purchased from Novus Biologicals (Littleton, CO, USA), β-actin, and horseradish peroxidase-goat anti-rabbit antibody were purchased from Beyotime Biotechnology (Shanghai, China).

**Table 1:** Primer sequences.

**Gene**	**Forward**	**Reverse**
AMPK	GGTGTACGGAAGGCAAAATGGC	CAGGATTCTTCCTTCGTACACGC
Sirt1	GGAGCAGATTAGTAAGCGGCTTG	GTTACTGCCACAGGAACTAGAGG
PGC-1α	AGCCTCTTTGCCCAGATCTCTT	GGCAATCCGTCTTCATCCAC
OPA1	TACCACAGTCCGGAAGAACC	ATTCGCCAAAACAGGACCAC
Mfn2	CCCTGCTCTTTTCTCGATGC	AGGTACCCTTTGACGTCCAG
Drp1	GCAACTGGAGAGGAATGCTG	CACAATCTAGCTGTTCTCGG
Fis1	AGATGGACTGGTAGGCATGG	CTACAGGGGTGCAGGAGAAA
β-actin	CATTGCTGACAGGATGCAGAAGG	TGCTGGAAGGTGGACAGTGAGG

### Preparation of septic acute kidney injury model and treatment

After one week of acclimation, mice were randomly divided into five groups (n = 8): control, LPS, LPS + PB2 (200 mg/kg, 100 mg/kg, and 50 mg/kg) groups. The LPS and LPS + PB2 groups were i.p. injected with LPS (10 mg/kg); the mice in the control group were received an equal volume of saline (i.p.). After LPS injection, the LPS + PB2 group mice were administered 200 mg/kg, 100 mg/kg, and 50 mg/kg of PB2 by gavage, respectively, whereas mice in the LPS group were administered an equal volume of solvent (saline) by gavage. At 24 h after LPS challenge, blood was collected from the abdominal aorta and kidney tissues were harvested. Briefly, the middle poles of the left kidney were fixed with carnoy fixative for further analysis such as pathological change, immunohistochemistry and immunofluorescence, the middle poles of the right kidney were embedded in optimal cutting temperature compound and flash frozen in liquid nitrogen, and the cortex of the upper poles of the right kidney that was close to the middle poles was separated and fixed in 3% glutaraldehyde for Transmission Electron Microscopy (TEM). Finally, the remaining kidney was flash frozen in liquid nitrogen and then stored at -80°C.

### Renal function evaluation

Blood samples were centrifuged for 10 min at 3000 rpm in 4°C to obtained serum. Creatinine and blood urea nitrogen levels were detected by the sarcosine oxidase method and urease method, respectively.

### Histopathology

Mouse kidneys were separated, and the middle poles of the left kidney were fixed in carnoy fixative, then dehydrated, embedded in paraffin, sectioned (thickness, 3 μm), and stained with hematoxylin-eosin staining (HE) and Periodic Acid-Schiff stain (PAS). Kidney injury was scored according to the Paller scoring method [74] (dilatation or flatness of renal tubules: 1 point; brush border injury: 1 point, shedding of brush border: 2 points; cast: 2 points, detached or necrotic cells in the lumen of the tubule: 1 point) by two pathologists. The total score was the sum of all scores in the renal tubular lumen.

### TUNEL assay

Apoptosis was detected by the TdT-mediated dUTP nick-end labeling (TUNEL) method accordance to the Deadend ^TM^ Fluorometric TUNEL system’s instructions. Briefly, the sections were deparaffinized, rehydrated by graded alcohol washes, and fixed in 4% formaldehyde. The tissue was then treated with recombinant Terminal Deoxynucleotidyl Transferase enzyme to form a polymeric tail on apoptotic cells and fluorescein was visualized using a Leica TCS SP5 confocal microscope (Wetzlar, Germany).

### Total RNA extraction and RT-PCR

Kidney tissue was disrupted in liquid nitrogen and total cellular RNA was extracted and converted to cDNA according to the manufacturer’s instructions. cDNA was amplified using a Roche Lightcycler 480 (Basel, Switzerland). Polymerase chain reaction (PCR) was carried out under the following conditions: 95°C, 30 s (1 cycle); 95°C, 5 s, 60°C, 20 s (40 cycles); 95°C, 5 s, 60°C, 60 s (1 cycle), and the relative expression of genes was normalized to that of β-actin.

### Western blotting

The proteins expression in the kidney was detected by western blotting. Briefly, the sample was lysed and clarified by centrifugation at 10,000 ×*g* for 10 min at 4°C. After denaturation, 30 μg of total proteins was separated by sodium dodecyl sulfate-polyacrylamide gel electrophoresis, transferred to polyvinylidene difluoride membrane, blocked with 5% bovine serum albumin, and sequentially incubated with the primary antibodies, followed by the secondary antibody and electrochemiluminescence by Azure Biosystems C500 (Azure Biosystems, Dublin, USA). The integrated optical density of the bands was analyzed using ImagePro Plus 6.0 software (Media Cybernetics, Rockville, MD, USA).

### Immunohistochemistry and immunofluorescence

The serial section was deparaffinized, rehydrated, and incubated with 3% H_2_O_2_, followed by blocking with 5% bovine serum albumin. For IHC, the sections were retrieved by citrate antigen retrieval solution before blocking, and then incubated with the NOX4 primary antibodies. Meanwhile, homotypic negative controls were processed in parallel with the corresponding IgG species in the corresponding dilutions, followed by the secondary antibody and immunostaining with DAB. For immunofluorescence analysis, the sections were incubated with LC3/COX IV or Pink1/COX IV antibodies in separately and secondary antibodies conjugated with Alexa Fluor to monitor mitophagy, and also paralleled with a homotypic negative control. The sections were counterstained with 4,6-diamidino-2 phenylindole, and their fluorescence signals were visualized using a Leica TCS SP5 confocal microscope. For the statistical analyses of integral optical density or fluorescence intensity, 10 random fields of the renal cortex within each section of 8 experimental kidneys from each group were counted. The overlap coefficient, Pearson's correlation, and co-localization fluorescent density of LC3/COX IV and Pink1/COX IV were analysis with Image J software (NIH, Bethesda, MD).

### Measurement of ROS levels

The level of superoxide (O^2-^) in kidney tissues was measured by staining the freshly frozen sections using DHE and MitoSox red. Briefly, the middle poles of the right kidney were embedded in optimal cutting temperature compound, flash frozen in liquid nitrogen, sectioned into 5 μm-thick slices, washed twice with prepared PBS, and incubated for 30 min with DHE (10 μmol/L) or MitoSox red for 10 min at 37°C. The resulting color reaction was immediately measured on a Leica TCS SP5 confocal microscope. The level of SOD, MDA, and reduced GSH in kidney lysis was measured by the hydroxylamine method, thiobarbituric acid method, and reductase recycling assay, respectively.

### Transmission electron microscopy

The cortex of the upper poles of the right kidney that was close to the middle poles was separated and fixed with 3% glutaraldehyde in 0.1 M sodium cacodylate buffer, infiltrated with 1% osmium tetroxide in cacodylate buffer, dehydrated, and infiltrated and embedded in eponate resin. The samples were then sectioned and stained with uranyl acetate and lead citrate, and images were collected using a Philips JEM-1400 transmission electron microscope (JEOL, Tokyo, Japan).

### Statistical analyses

The statistical analyses were performed using SPSS statistical software (SPSS 20.0, USA). To compare the differences among the groups, a one-way analysis of variance (ANOVA) was used for normally distributed data, and data was expressed as means ± standard deviation. Kruskal-Wallis test was used for non-normally distributed data, and data was expressed as the median. The rank-sum test was used to identify pathological damage. The results with P-values of ≤ 0.05 were considered as statistically significant.
